# A Microfluidic Lab-on-a-Disc (LOD) for Antioxidant Activities of Plant Extracts

**DOI:** 10.3390/mi9040140

**Published:** 2018-03-21

**Authors:** Nurhaslina Abd Rahman, Fatimah Ibrahim, Mohammad M. Aeinehvand, Rohana Yusof, Marc Madou

**Affiliations:** 1 Department of Biomedical Engineering, Faculty of Engineering, University of Malaya, 50603 Kuala Lumpur, Malaysia; haslin42@siswa.um.edu.my; 2 Centre for Innovation in Medical Engineering, Faculty of Engineering, University of Malaya, 50603 Kuala Lumpur, Malaysia; m.aeinehvand@itesm.mx (M.M.A.); mmadou@uci.edu (M.M.); 3 School of Engineering and Sciences, Tecnologico de Monterrey, Av. Eugenio Garza Sada 2501 Sur, 64849 Monterrey, NL, Mexico; 4 Department of Molecular Medicine, Faculty of Medicine, University of Malaya, 50603 Kuala Lumpur, Malaysia; rohana@um.edu.my; 5 Department of Biomedical Engineering, University of California, Irvine, CA 92697, USA

**Keywords:** Lab-on-a-Disc (LoD), centrifugal microfluidic CD, plant antioxidant activity, antioxidants, DPPH

## Abstract

Antioxidants are an important substance that can fight the deterioration of free radicals and can easily oxidize when exposed to light. There are many methods to measure the antioxidant activity in a biological sample, for example 2,2-diphenyl-1-picrylhydrazyl (DPPH) antioxidant activity test, which is one of the simplest methods used. Despite its simplicity, the organic solvent that has been used to dilute DPPH is easily evaporated and degraded with respect to light exposure and time. Thus, it needs to be used at the earliest convenient time prior to the experiment. To overcome this issue, a rapid and close system for antioxidant activity is required. In this paper, we introduced the Lab-on-a-Disc (LoD) method that integrates the DPPH antioxidant activity test on a microfluidic compact disc (CD). We used ascorbic acid, quercetin, *Areca catechu*, *Polygonum minus*, and *Syzygium polyanthum* plant extracts to compare the results of our proposed LoD method with the conventional method. Contrasted to the arduous laborious conventional method, our proposed method offer rapid analysis and simple determination of antioxidant. This proposed LoD method for antioxidant activity in plants would be a platform for the further development of antioxidant assay.

## 1. Introduction

Free radicals are atoms with unpaired valence electrons that cause its chemical instability and reactivity. The valence electron of one atom accepts an electron from another molecule, which forms a new free radical. This process repeats itself, creating a free radical cascade. When free radical production becomes excessive in the body, it may cause oxidative stress, a condition whereby the body cannot counter-attack the production of free radicals, leading to cellular damage and cell death [1]. Free radicals have been implicated in many diseases, such as diabetes, hypertension, Parkinson’s, Alzheimer’s, and heart diseases [2,3,4,5,6]. Additionally, free radical production increases as the individual gets older, thereby accelerating the aging process [7].

Antioxidants are molecules that can donate electrons to stabilize free radical species. They play many important roles in daily life. For example, antioxidants are hypothesized to prevent the deleterious effects of free radicals on cells [8]. In the medical field, antioxidants have been used as an alternative disease therapy for diabetes mellitus, reperfusion injury, and inflammatory diseases. They also help to prevent atherosclerosis and carcinogenesis in the human body [9]. In the food industry, antioxidants can prevent the deterioration of food constituents and prevent it from spoiling. In addition, antioxidants absorb ultraviolet (UV) radiation, which minimizes the risk of skin cancer [10]. There are many potential natural chemical constituents that can be exploited from plants, such as medical plants containing high antioxidant properties, which are scientifically significant in the human body [11].

There are many methods to determine the antioxidant properties in a sample. Each of the methods work differently based on the chemical mechanism, among which, the 2,2-diphenyl-1-picrylhydrazyl (DPPH) antioxidant activity test is the most common and easiest method used to determine the antioxidant activity in a sample [12]. The DPPH antioxidant assay has been used widely to determine various antioxidant activities in food, beverages, and plants. Furthermore, it is also used to evaluate the antioxidant activity of nutrients in the human body [8]. DPPH is a stable, deep purple free radical powder, which will change to a pale yellow after reacting with the antioxidant. The free radical DPPH will react with the antioxidant, which acts as a hydrogen donor that stabilizes DPPH.

Despite the simplicity of the DPPH antioxidant assay, DPPH itself is easily decomposed over time and is highly sensitive to changes in temperature, pH, and light exposure [13,14]. The DPPH solutions need to be diluted in organic solvent and prepared fresh before running the antioxidant activity test. According to Deng et al. [15], DPPH solution needs to be used within two hours of preparation to prevent the decomposition of the DPPH solution. It is also reported that the DPPH absorbance decreases within 90 min with changes in temperature. The absorbance of DPPH solution will also decrease when exposed to light within two hours, while in the dark, there are no significant changes in DPPH absorbance.

Many fundamental bio-assay methods are carried out manually. The experimental steps are often time-consuming and require expert skills. The amalgamation of human-prone error, especially in the pre-analytical phase, may also cause incorrect interpretations or false positive results in the analytical and post-analytical phases [16]. One of the most common pre-analytical errors is caused by pipetting error. The pipetting steps are the most common repetitive tasks for laboratorians; however, this step is often taken for granted. The pipetting steps, especially the microscale volumes, are very crucial and accuracy must be ensured to prevent erroneous results. Additionally, organic solvents evaporate easily and may contribute to volume loss. Errors also happen due to the repetitive steps and duplications when laboratorians are handling large amounts of samples.

Lab-on-a-Disc (LoD), which are also known as centrifugal microfluidic, are a part of the microfluidic device in the form of a disc with a spinning motor. LoD is a part of the Micro Total Analysis System (µTAS), which offers miniaturization and automation of most of the chemical and biological analysis systems. LoD offers many advantages, such as simplicity of the assay procedure, fast results, and cost efficiency. LoD has automated many bioassays, such as Enzyme-linked Immunosorbent Assay (ELISA) and the loop-mediated isothermal amplification (LAMP) assay [17,18]. When compared to lab-on-a-chips (LoCs) that require syringe pumps, LoD runs in a closed system and has an “all-in-one disc” assay procedure. LoD decreases the amount of reagent, sample usage and total processing time during the experiments. Subsequently, it also offers parallel or sequential loading of solutions. LoD skips multiple sample preparations and pipetting steps, which reduces human-prone handling error.

Jungwoo et al. [8] have demonstrated an LoC platform to evaluate the liver metabolism of antioxidants in food. He is focusing on mimicking the human liver metabolism and the detection of subsequent metabolized antioxidant food components. The design consist of a two microfluidic compartment; the first part contain human liver enzyme that mimic liver metabolism and the second part is DPPH detection of antioxidant activity of food components. In his design, the liver enzyme fractions were immobilized and the reaction with the DPPH solution were tested. Meanwhile, Xuhua et al. have used hydrogen peroxide techniques on a chip and to screen the antioxidant capacity by using herbal extracts [19]. His focused is more on the fabrication of the chip by using the thin-film organic photodiodes and the chemiluminescence detection of the antioxidant in the herbal extracts.

In this paper, we present an approach of LoD method for antioxidant plant activities. We offer automation and sample miniaturization of the DPPH antioxidant activity test with parallel sequential sample loading and mixing. Our proposed LoD method has been tested on ascorbic acid, quercetin, *Areca catechu*, *Polygonus minus*, and *Syzygium polyanthum* plant extract.

## 2. Materials and Methods

### 2.1. Plant Materials Preparation

The plants *A. catechu*, *P. minus* and *S. polyanthum* were collected from Selangor and Perak, Malaysia. The plants were chosen based on their reported high antioxidant activity among local plant species [20,21,22]. The plants were dried in a laboratory oven at 40 °C until crisp, then pulverized into a powder using a mechanical grinder. The ground powder was then soaked and shaken in 95% ethanol for 48 h. The solvent mixture was then filtered using Whatman No. 1 filter paper to eliminate the plant debris. After this, the excess solvents were eliminated using a rotary evaporator (Buchi Rotavapor R-114, Büchi Labortechnik AG, Flawil, Swizerland), then freeze-dried, and stored for 20 °C until further use in the experiment.

### 2.2. DPPH Conventional Antioxidant Activity Test

The antioxidant activity of the samples were determined using the method described by Saha et al. [23], with some modification. The deep purple colorimetry reduction of DPPH was determined by using a spectrophotometer (Epoch, BioTek Instruments, Winooski, VT, USA) at 517 nm with three repeated measurements. First, the plant extracts were diluted to four final concentrations of 25, 50, 75, and 100 mg/mL, to make 1 mL sample solutions in ethanol. Then, 1 mM of DPPH in methanol was prepared and added to the test solution and left to incubate for 30 min at room temperature. For the control experiment, ethanol was added with DPPH. Finally, the absorbance values were measured and the antioxidant activities were calculated using the following equation.

(1) $$Antioxidant\;activity\;=\frac{Control-Test\;Sample}{Control}$$

### 2.3. Microfluidic Compact Disc (CD) Design and Fabrication

**Figure 1:**
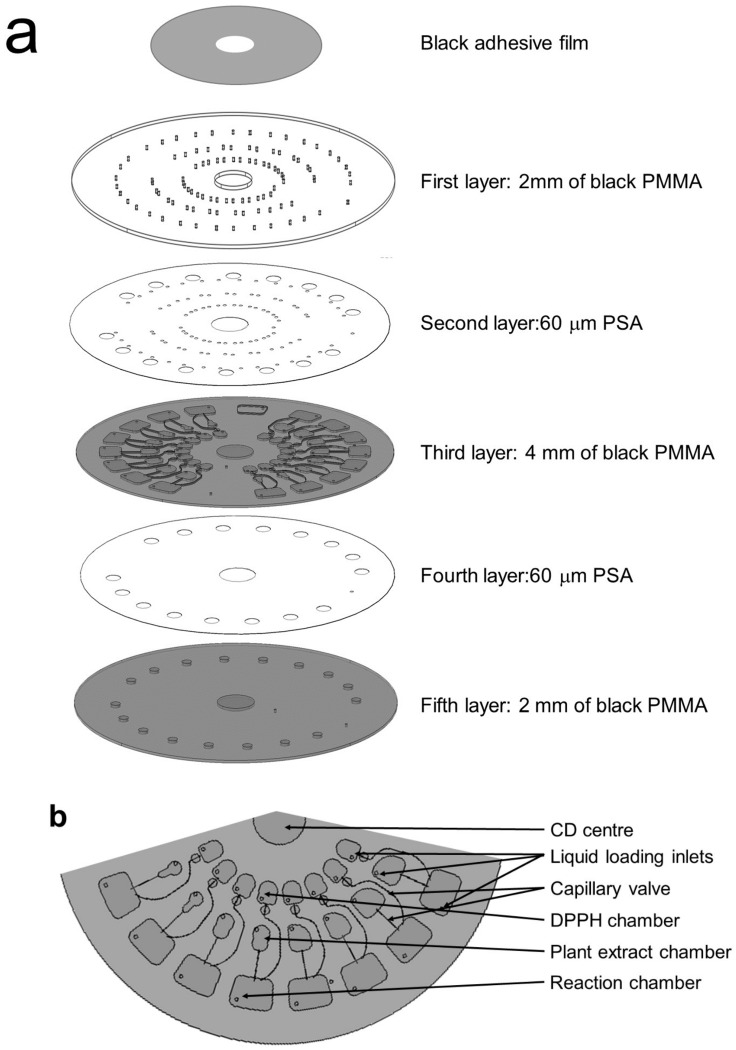

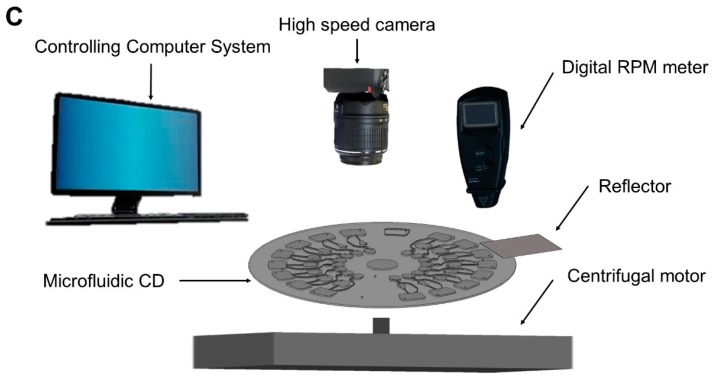
(**a**) An exploded view of the photo-protective layers of the microfluidic compact disc (CD). The CD is consist of five layers. The first, third, and the fifth layer was made from black PMMA layer. While the second and the third layer was made from PSA; (**b**) A top view of the third layer of the antioxidant microfluidic CD with different specification of chamber. The plant extract chamber was varied in four different sizes and duplicated for two consecutive concentrations; (**c**) The whole experimental set up. The whole experiment set up consists of the computer controlling systems and the custom Lab-on-a-Disc (LOD) spinning test system. The centrifugal motor and the high-speed camera were connected to the computer to control the CD speed (rpm) and visualisation. The reflector will give the signal to the digital RPM.

The photoprotective microfluidic was designed using computer-aided design software (AutoCAD), as shown in Figure 1a. The photoprotective microfluidic CD consists of five layers: three black Polymethyl Methacrylate (PMMA) and two pressure sensitive adhesive (PSA) layers. The first layer is a transparent PMMA disc and contains injection/venting holes. A black adhesive film is used to cover the PMMA disc except the reaction chambers. The second layer is PSA and the third layer is a black PMMA disc with the microfluidic channel and chamber features engraved on it. The fourth layer is PSA and the fifth layer of the microfluidic CD is made of black PMMA. The CD features details can be seen in the Supplementary section.

All of the engraving of microfluidic channel features was done using a Computer Numerical Control (CNC) machine VISION 2525 by Vision Engraving and Routing Systems, USA, as shown in Figure 1b. The engraved microfluidic channel features three chambers: a DPPH chamber, plant extract chamber, and reaction chamber. The DPPH chamber was connected to the valve to control the flow of DPPH solution to the reaction chamber. The inlet holes and microfluidic features in the PSA layers were cut using a cutter plotter machine (GCC P2-60/PUMA II, by GCC, Taiwan). Each layer of microfluidic CD is press-bonded together using a custom-made pressing tool.

Figure 1c shows the whole experimental set up, which consists of computer controlling systems and custom LoD spinning test systems. The centrifugal motor and the high-speed camera were connected to the computer to control the microfluidic CD rotating speed (rpm) and visualization, respectively. The reflector attached to the microfluidic CD will provide the signal to the digital RPM meter to determine the CD’s rotation speed.

### 2.4. Integrated Microfluidic DPPH CD Operations

After preparing the plant extract (as discussed in the previous section), the plant extracts along with the two-standard reagent of antioxidant (ascorbic acid and quercetin) were then preloaded into the plant extract chambers. The plant extract chambers in the middle were designed in four different sizes in duplicate for 25, 50, 75, and 100 mg/mL plant extract. Meanwhile, as shown in Figure 1b, the reaction chamber was located at the bottom of the microfluidic CD and prefilled with the solvents. It is the final chamber for the mixture of the solvent, plant extract, and the DPPH. The DPPH chamber connected with a valve was designed to occupy 1 mM DPPH. After all of the reagents are preloaded to the designated chamber, the holes were sealed with PCR sealing tapes. The centrifugal microfluidic process starts after all the liquids have been loaded in to the designated chamber. The spinning operation is summarized in Table 1. The spinning is stopped after 30 min and the absorbance reading were measured.

### 2.5. Absorbance Reading

There are two devices used to read the antioxidant activity absorbance, which are a microplate reader and the CD reader. The microplate reader was used to compare the antioxidant absorbance activity for both the conventional and LoD method reading over 5 min intervals up to 30 min. The final solutions obtained from the conventional and LoD reaction methods were then transferred to the 96 well plates for the absorbance measurements.

**Figure 2:**
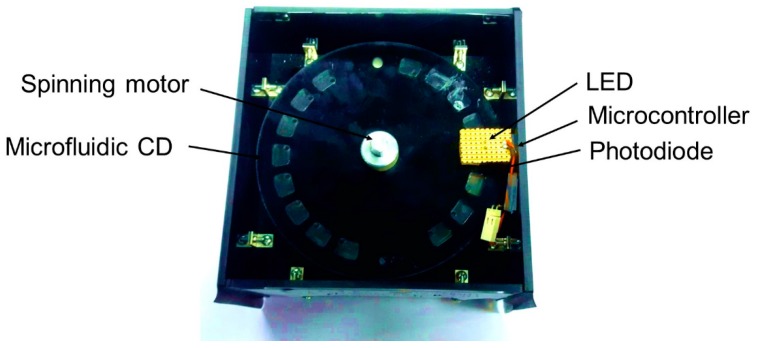
The CD reader is used to read the final absorbance directly from the CD. The results were then compared to the microplate reader.

Whereas, the CD reader (Figure 2) was used to measure the absorbance of the final biochemical solutions in the microfluidic CD 24, 25. The CD reader consisted of a 517 nm LED and photodiode to measure the light intensity passing through the solution. A microcontroller (Atmega328PU, Microchip Technology Inc., Chandler, AZ, USA) connected with the photodiode, calculates the absorbance of the light passing through the solutions. A stepper motor, a Hall Effect sensor and a magnet help to incorporate the alignment among the reaction chamber, LED, and photodiode. The CD reader structure was built using black PMMA to reduce the optical noise.

## 3. Results and Discussion

### 3.1. Microfluidic CD Operations

**Figure 3:**
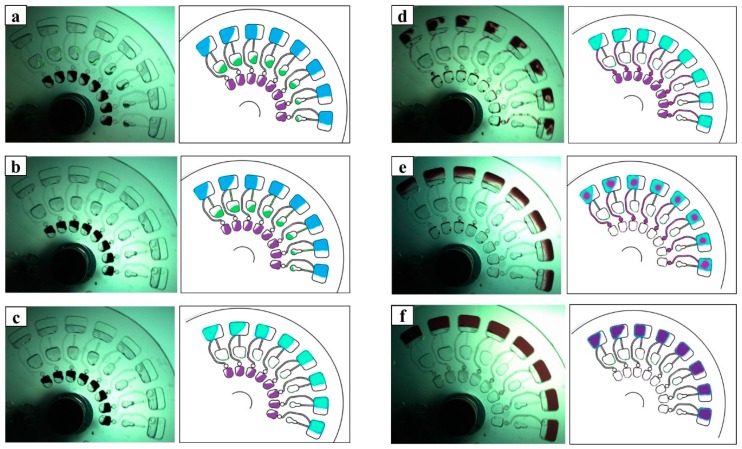
Entire sequences of the images and schematic illustrations in the microfluidics CD. (**a**) The initial of the experiment, speed 0. (**b**) The plant extract solution started to flow into the capillary valve; speed has been increased from 0 to 300 rpm. (**c**) The plant extract chambers have been emptied; speed 300 rpm. (**d**) DPPH solution flow out from its chamber to the capillary valve; speed has been increased slowly from 300 to 800 rpm. (**e**) The DPPH chamber was emptied; speed 800 rpm. (**f**) All of the solutions were mixed properly in the reaction chamber; speed 1400 rpm.

Figure 3 shows the actual images from the complete process of microfluidic CD operations, sequential loading, and mixing. For display purposes, the microfluidic CD was fabricated using clear PMMA. The schematic illustrations on the left side are drawn to show and explain the experiments, with corresponding images captures from the video of the high speed camera. In the first step, Figure 3a has shown the liquids have been preloaded into the designated chambers i.e., DPPH solution in the DPPH chamber, plant extract solution in the plant extract chamber, and solvents in the reaction chamber. To start the experiment, the speed of the centrifugal motor was increased slowly. During this process, the plant extract solution started to flow into the capillary valve. This process is shown in Figure 3b. Subsequently, after the rotation speeds have reached 300 rpm, all the plant extract chambers have been emptied and the solution has been mixed in the reaction chamber with the solvent (as shown in Figure 3c). In Figure 3d, the speed was increased, which makes the DPPH solution flow out from its chamber to the capillary valve. At 800 rpm, it was noticed that all the DPPH solution was emptied from the chamber as the solution moved to the reaction chamber, which is shown in Figure 3e. Finally, in Figure 3f the centrifugal speed was increased to 1400 rpm to ensure all the solutions were mixed properly.

Our proposed method has minimized human operations in the DPPH antioxidant test, the repetitive pipetting, loading, and mixing steps can be skipped by flow control mechanism of the microfluidic CD. Basically, in our microfluidic CD design, the capillary passive valves were used to control the sequential fluidic flow and the mixing of the liquids. The manipulation of the chamber position, centrifugal force and the capillary valve have determined the sequences of fluids flows to the reaction chamber, the details and analysis of the valves burst frequency is described by Thio et al. [24] and Kazemzadeh et al. [25].

Thio et al. and Kazemzadeh et al. in their paper have been discussed extensively about the theory of the liquid flows inside the passive capillary valves toward the target chamber (in this paper is reaction chamber). In order of the liquid to move from one chamber to another, the capillary pressure need to be overcome by increasing the speed of the motor (centrifugal pressure will have increased parallelly). In this paper, the location and the geometry of the plant extract chamber and the DPPH chamber on the microfluidic CD have enable the liquid flows manipulation into the reaction chamber.

### 3.2. Comparisons of Conventional and LoD DPPH Antioxidant Activity Method

In this section, the comparison results of the conventional and LoD DPPH antioxidant methods are shown and discussed. All of the samples were tested for antioxidant activity by using LoD method and compared with the conventional DPPH antioxidant activity test for 30 min as this is the standard time and common procedures practises in the conventional DPPH assay [26]. Subsequently, the results were further analyzed in 5 min intervals up to 30 min. For the purposes of analysis, a repeated ANOVA measure by IBM SPSS statistical software version 24 was used.

**Figure 4:**
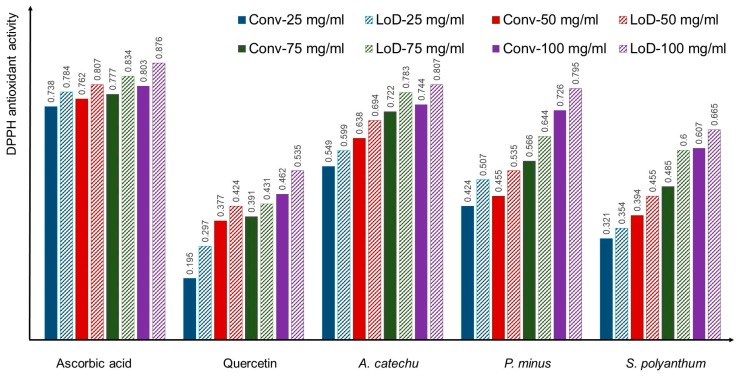
Comparison of antioxidant activity in conventional and LoD method at different concentrations.

In Figure 4, the comparisons of the ascorbic acid, quercetin, *A. catechu*, *P. minus*, and *S. polyanthum* activity antioxidant has been presented between conventional and LoD method at each concentration. The results showed that there was a significant difference of * *p* ≤ 0.05 between the conventional and LoD method at each concentration. What can be clearly observed in Figure 4 is the levels of the DPPH activity in the LoD method are consistently higher than the conventional method. In the conventional method, the reaction is let to stand for 30 min without any force being applied on it. Whereas, in the LoD method, mixing scheme has been applied which involves the combination of centrifugal force, Euler force, and Coriolis force. Centrifugal and Euler force is important for the automated liquid handling processing. While Coriolis force creates the stirring effect in the reaction chamber that leads to better liquid homogenization and diffusion of the particles.

Efficacious sample mixing accelerate the chemical reaction and decrease the time of assay [27]. In a chemical reaction, the ability to create a fast homogenous reactant mixture is crucial, especially in the small sample volume. For rapid chemical reactions to occur, a fast kinetics reaction and high contact frequency between particles can be increased with a good mixing efficiency, which this phenomena has been provided in the LoD platform [28,29]. Our proposed method has introduced a passive mixers which maximize the area of the chemical reaction to occur in the reaction chamber. With the constant application of Coriolis stirring effect by the CD rotation, the chemical reaction between the plant extract and the DPPH has been enhanced, which in contrast does not happen in the conventional method.

#### 3.2.1. Ascorbic Acid

**Figure 5:**
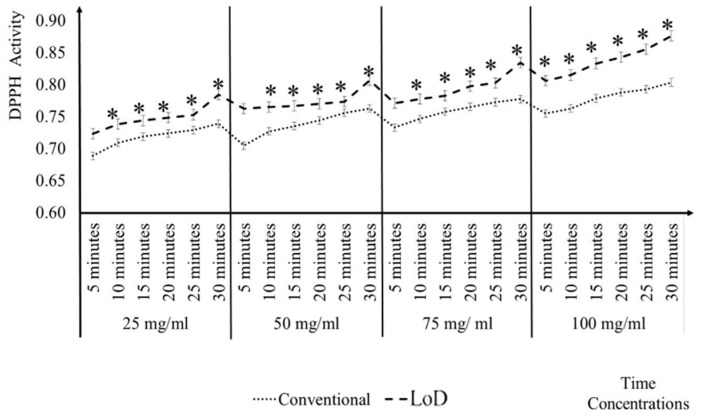
Comparison of ascorbic acid antioxidant activity in the conventional and LoD method by 5 min interval.

In Figure 5, it can be seen that, the DPPH activity value with the LoD method at 10 min gave the same activity level as the conventional method for 25 mg/mL, 50 mg/mL, and 75 mg/mL ascorbic acid at 30 min. On the other hand, 100 mg/mL with the LoD method of ascorbic acid took only 5 min to reach the antioxidant activity level of conventional method equal to 30 min. On average, the LoD method that was tested with ascorbic acid was able to produce similar measurements when compared to the conventional method with an analysis reduction time of 21.25 min i.e., the LoD method duration was 5 min at 100 mg/mL, whereas the conventional method took 30 min. The mean, SE, and SD for the different concentrations and different time intervals were clearly seen in Appendix A. The small values of the SE and SD indicate that the results are repeatable and precision of the mean value [30].

#### 3.2.2. Quercetin

**Figure 6:**
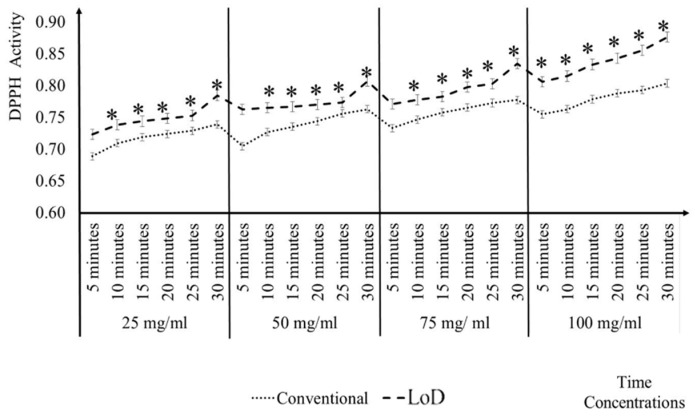
Comparison of quercetin antioxidant activity determined using conventional and LoD methods by 5 min time intervals.

Figure 6 shows that, its takes 20 min for 25 mg/mL and 15 min for 50 mg/mL and 75 mg/mL of Quercetin to give the comparable antioxidant activity level of the DPPH conventional methods at 30 min. However, for 100 mg/mL Quercetin, it only took 10 min to give the same antioxidant activity level as the DPPH conventional methods at 30 min. On average, the LoD method accelerated the analysis time for Quercetin by 15 min when compared to the conventional methods at 30 min. The mean, SE, and SD for the different concentrations and different time intervals can be clearly seen in Appendix B.

#### 3.2.3. *A. catechu*

**Figure 7:**
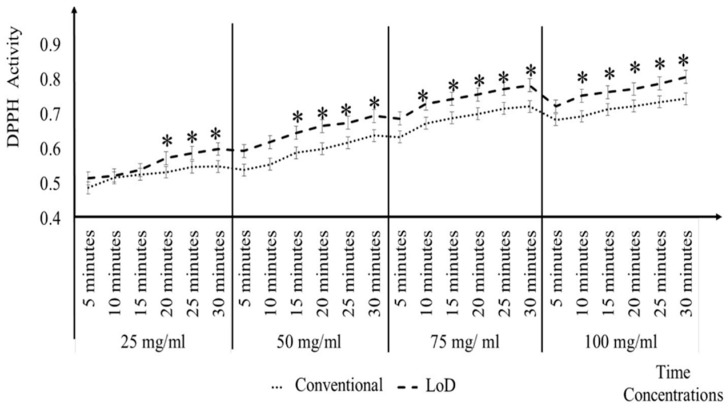
Comparison of *A. catechu* antioxidant activity determined using conventional and LoD methods by 5 min time interval.

Figure 7 shows that it took 20 min and 15 min for 25 mg/mL and 50 mg/mL, respectively, to give the same antioxidant activity level as the DPPH conventional methods at 30 min. Meanwhile, for 75 mg/mL and 100 mg/mL, at 10 min the LoD method was sufficient to give the same antioxidant activity as the DPPH conventional methods at 30 min. On average, the LoD method that was tested with *A. catechu* plant extract gave a result that was 16.25 min faster when compared to the conventional methods at 30 min. The analysis can be seen in Appendix C.

#### 3.2.4. *P. minus*

**Figure 8:**
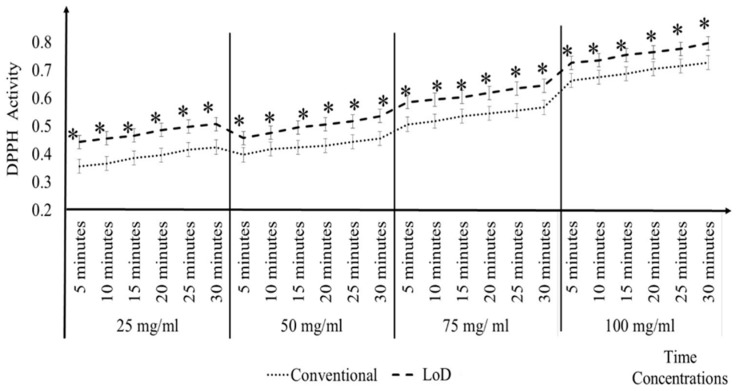
Comparison of *P. minus* antioxidant activity determined using conventional and LoD methods 5 min time interval in each concentration.

The results in Figure 8 shows that 5 min for 25 mg/mL, 50 mg/mL, 75 mg/mL, and 100 mg/mL in the LOD method are sufficient to give the same antioxidant activity as the same concentrations at 30 min for the DPPH conventional methods. The analysis of the mean, SE and SD for different concentrations and different time intervals can be clearly seen in Appendix D.

#### 3.2.5. *S. polyanthum*

**Figure 9:**
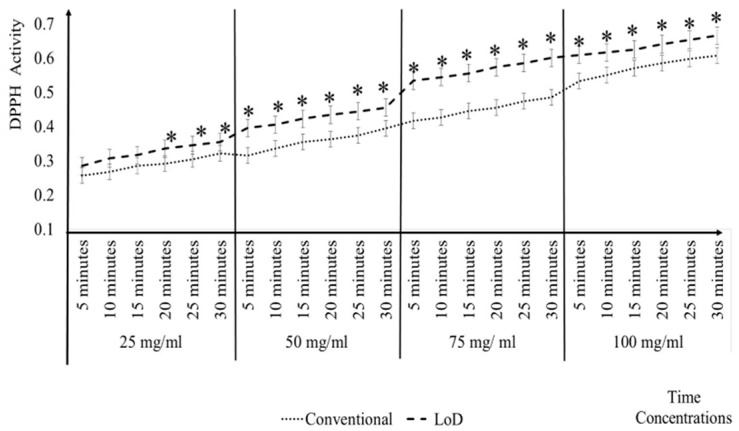
Comparison of *S. polyanthum* antioxidant activity in conventional and LoD method by 5 min time interval.

The results in Figure 9 indicate that the LoD method was able to give the same antioxidant level as early as 5 min at 50 mg/mL, 75 mg/mL, and 100 mg/mL. By using the LoD method, it is believed with the increase of the concentrations, the time to reach the same activity level as the conventional method will be decreased. On average, the LoD method that was tested with *S. polyanthum* plant extract gave a result that was 21.25 min faster when compared to the conventional methods. Refer Appendix E for the analysis.

The results has been shown in Figure 4, Figure 5, Figure 6, Figure 7 and Figure 8, where the reading does not reach plateau state in the microfluidics CD method, in contrast to the finding with Phonchai et al., which have shown a plateau DPPH activity reading with respective mixing time [31]. However, in contrast many researchers have also agreed that the kinetics reaction in DPPH assay may persist from minutes to hours. Although it is recommendable to allow for the reaction and measurement until the plateau reading state, the diverse completion kinetic reaction time in DPPH have led us to use 30 min as a single standard measuring time [26,32,33]. Additionally, Xie et al. [13] have illustrated that, regardless of the incubation time, the assay definitely reflects the stoichiometry DPPH reaction with the antioxidant in the sample.

Referring to the Figure 5, Figure 6, Figure 7, Figure 8 and Figure 9, it can be clearly seen that the time taken to measure the DPPH activity varies among different samples. For example, the analysis of 25 mg/mL solutions of ascorbic acid and quercetin using LoD method was able to give the comparable DPPH antioxidant activity to the conventional method at 10 and 20 min, respectively, meanwhile the LoD method reduced the time that was taken to analyse *P. minus* to 5 min, when compared to the standard 30 min of the conventional method. This result may be explained by the fact that the purity, compound mixtures, different form, and weight of molecules that are present in a sample did play a role for diverse antioxidant activity. In this study, the plant extract have been used to test the LoD method, the presence of compound, such as phenol, may cause interference and affect the reading when compared to the actual activity, which expound the different activity time between each plant [34,35].

**Figure 10:**
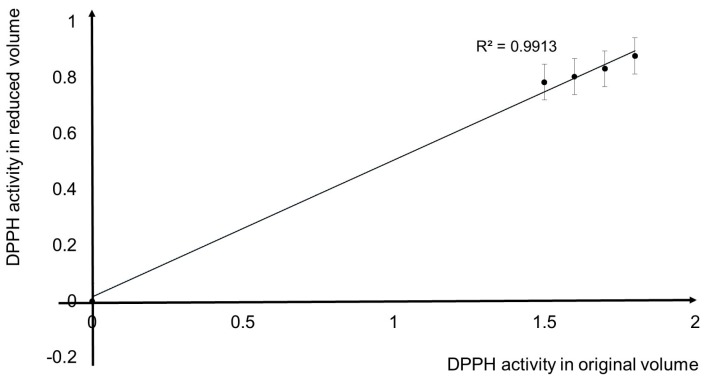
DPPH activity correlation between the original and reduced volume in proposed method.

The conventional DPPH method use a high concentrations of the plant extract (i.e., 100, 200, 400, 800 mg/mL), which proportionally need large reagent volumes. The proposed method reduced the volume usage to the final of 200 µL to provide a good range of detection as the conventional method [31]. Figure 10 shows a fitted correlation of the original conventional method and the proposed method reduced volume. The decreased amount of reagent and sample that were used in the experiments offer optimal advantages for the rare samples and limited reagent supply.

### 3.3. Comparisons of CD Reader and the Conventional Microplate Reader

**Figure 11:**
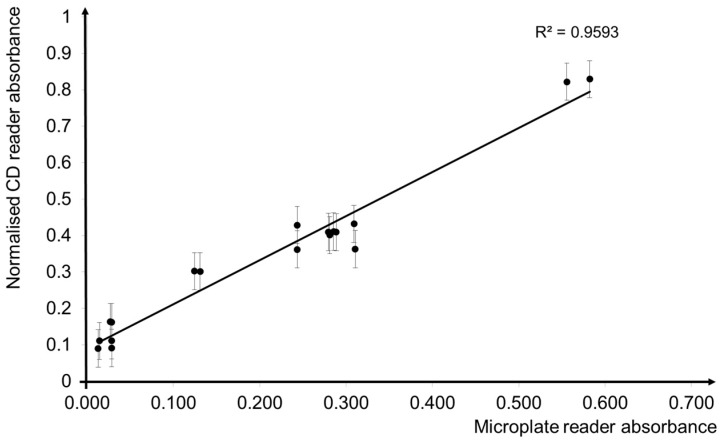
Graph of coefficient correlation between the CD plate reader and the microplate reader.

The experimental work was carried out with a modified version of custom-made CD spin test system [36,37]. The readings were taken after a 30 min incubation for both the proposed LoD method and the conventional DPPH method. The data in Figure 11 shows a fitted coefficient and correlations (r^2^ = 0.96) between the reading from the CD reader and the microplate reader, indicating a close relationship between the CD reader and the microplate reader. Unlike the conventional method, the CD reader can read the absorbance of the entire sample directly from the microfluidic CD. It is designed specially to work in complement with the microfluidic CD, and the pipetting process of transferring the solutions to the 96 well plate has be eliminated. The process of transferring the solutions to the 96 well plate risks losing volume, which can be rectified by a closed and automated system, such as that used in the LoD platform. This CD reader endpoint reading system complete and support to the development of an integrated platform for the antioxidant microfluidics CD system.

## 4. Conclusions

In this study, we have integrated the conventional DPPH antioxidant activity test by proposing a microfluidics CD method for the detection of antioxidant activities in plants. The sequential sample loading and mixing in a closed LoD system minimizes human error and volume loss due to manual pipetting procedures. The “load and run concept” in the proposed LoD method has omitted the repetitive pipetting, mixing, and loading steps in the DPPH conventional method. With four parallel concentrations and duplications that able been run simultaneously on the microfluidic CD, this study has shown that the proposed LoD method for antioxidant activities of the plant extracts procedures has been automated. Using this LoD technique not only it automates the processes but also helps to reduce the incubation time to five minutes, to reach the same antioxidant activity level as the conventional method of 30 min. This approach would work as an application for antioxidant activity and will act as a platform for the determination of future antioxidant activity in a plants or another type of sample.

## Figures and Tables

**Table 1:** Spinning program of the microfluidic CD.

Step	Spinning Speed (rpm)	Time *	Spinning Direction	Process
1	0	1 min	nil	Sample preloading
2	300	30 s	Clockwise	Plant extracts flowing to the reaction chamber and emptied
3	800	15 s	Anticlockwise	2,2-diphenyl-1-picrylhydrazyl (DPPH) solution flow out from the DPPH chamber to the capillary valve and reached reaction chamber
4	1400	5 min interval to 30 min	Clockwise and anticlockwise	The disc is rotated for mixing purposes

* The time includes the acceleration time to reach the target speed and to change the rotation (2 s).

## Supplementary Materials

The following are available online at http://www.mdpi.com/2072-666X/9/4/140/s1.
