# Supplementary material for: A Microfluidic Lab-on-a-Disc (LOD) for Antioxidant Activities of Plant Extracts
Source: Micromachines (Basel). 2018 Mar 21;9(4):140. doi: 10.3390/mi9040140 (PMC6187507; doi:10.3390/mi9040140)
Supplement: Supplementary file 1 [file micromachines-09-00140-s001.zip › Supplementary information.docx]

Supplementary information

CD features


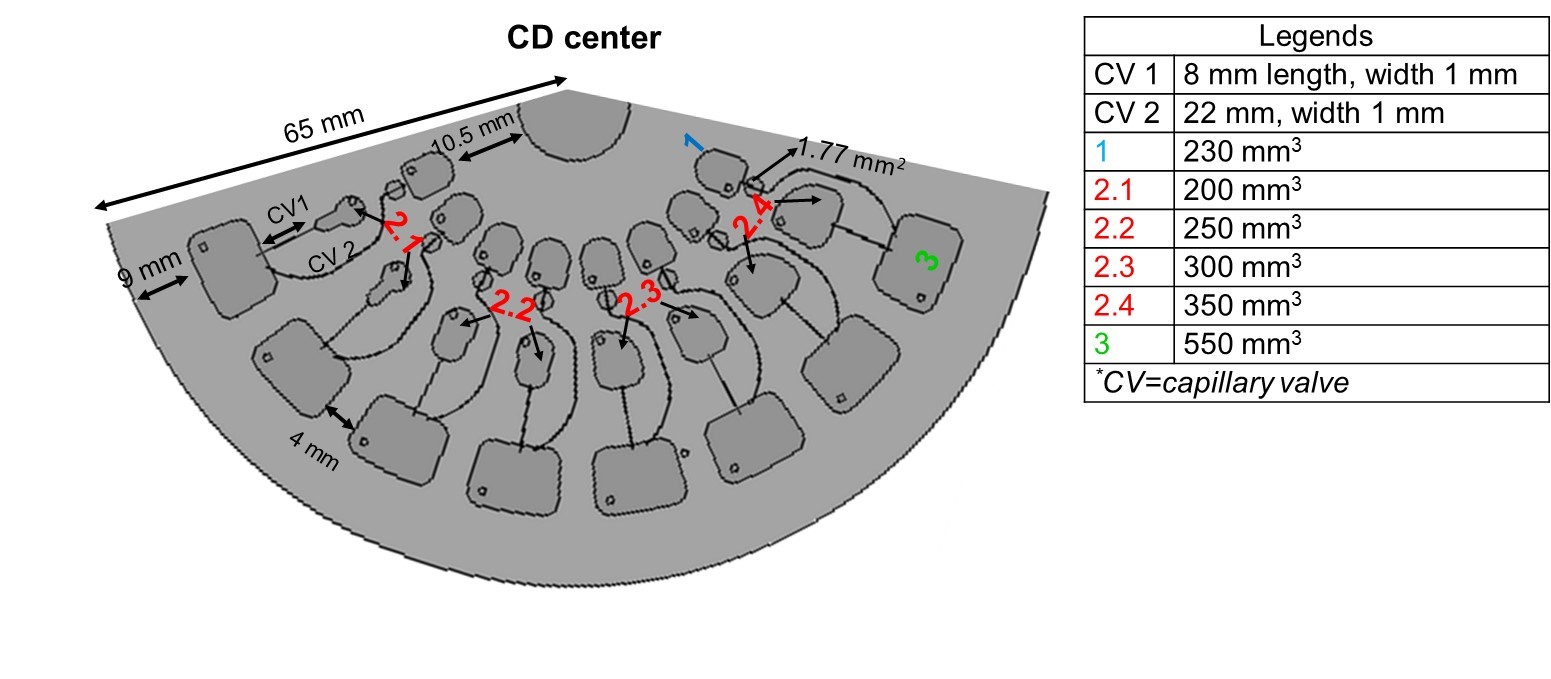


Appendix A

Mean, standard error and the standard deviation of ascorbic acid antioxidant activity

| Concentrations | | 5 minutes | | 10 minutes | | 15 minutes | | 20 minutes | | 25 minutes | | 30 minutes | | F  value | P value |
| --- | --- | --- | --- | --- | --- | --- | --- | --- | --- | --- | --- | --- | --- | --- | --- |
|  |  | Con | LoD | Con | LoD | Con | LoD | Con | LoD | Con | LoD | Con | LoD |  |  |
| 25  mg/ml | Mean | 0.688 | 0.723 | 0.709 | 0.738 | 0.718 | 0.743 | 0.723 | 0.748 | 0.728 | 0.753 | 0.738 | 0.784 | 93.332 | p≤0.05 |
|  | SE | 0.001 | 0.001 | 0.000 | 0.001 | 0.001 | 0.001 | 0.001 | 0.001 | 0.001 | 0.001 | 0.001 | 0.001 |  |  |
|  | SD | 0.001 | 0.002 | 0.001 | 0.002 | 0.001 | 0.001 | 0.001 | 0.001 | 0.001 | 0.001 | 0.001 | 0.001 |  |  |
| 50  mg/ml | Mean | 0.704 | 0.760 | 0.726 | 0.765 | 0.735 | 0.767 | 0.744 | 0.769 | 0.756 | 0.773 | 0.762 | 0.807 |  |  |
|  | SE | 0.001 | 0.001 | 0.001 | 0.001 | 0.001 | 0.001 | 0.001 | 0.001 | 0.001 | 0.001 | 0.001 | 0.001 |  |  |
|  | SD | 0.001 | 0.001 | 0.001 | 0.001 | 0.001 | 0.001 | 0.001 | 0.002 | 0.001 | 0.001 | 0.001 | 0.002 |  |  |
| 75  mg/ml | Mean | 0.733 | 0.771 | 0.746 | 0.777 | 0.758 | 0.782 | 0.765 | 0.797 | 0.772 | 0.802 | 0.777 | 0.834 |  |  |
|  | SE | 0.001 | 0.001 | 0.001 | 0.001 | 0.001 | 0.001 | 0.001 | 0.001 | 0.001 | 0.001 | 0.001 | 0.001 |  |  |
|  | SD | 0.002 | 0.001 | 0.001 | 0.001 | 0.001 | 0.001 | 0.001 | 0.001 | 0.001 | 0.001 | 0.002 | 0.001 |  |  |
| 100mg/ml | Mean | 0.754 | 0.806 | 0.762 | 0.814 | 0.778 | 0.833 | 0.787 | 0.842 | 0.792 | 0.855 | 0.803 | 0.876 |  |  |
|  | SE | 0.001 | 0.001 | 0.001 | 0.001 | 0.001 | 0.001 | 0.001 | 0.001 | 0.001 | 0.001 | 0.001 | 0.001 |  |  |
|  | SD | 0.002 | 0.002 | 0.001 | 0.001 | 0.002 | 0.002 | 0.001 | 0.001 | 0.001 | 0.001 | 0.001 | 0.001 |  |  |

Appendix B

Mean, standard error and the standard deviation of quercetin antioxidant activity

| Concentrations | | 5 minutes | | 10 minutes | | 15 minutes | | 20 minutes | | 25 minutes | | 30 minutes | | F  value | P value |
| --- | --- | --- | --- | --- | --- | --- | --- | --- | --- | --- | --- | --- | --- | --- | --- |
|  |  | Con | LoD | Con | LoD | Con | LoD | Con | LoD | Con | LoD | Con | LoD |  |  |
| 25  mg/ml | Mean | 0.114 | 0.154 | 0.126 | 0.173 | 0.138 | 0.185 | 0.153 | 0.204 | 0.183 | 0.214 | 0.195 | 0.297 | 82.495 | p≤0.05 |
|  | SE | 0.001 | 0.001 | 0.001 | 0.001 | 0.001 | 0.001 | 0.001 | 0.001 | 0.001 | 0.001 | 0.001 | 0.001 |  |  |
|  | SD | 0.001 | 0.002 | 0.001 | 0.001 | 0.001 | 0.001 | 0.001 | 0.001 | 0.001 | 0.001 | 0.001 | 0.001 |  |  |
| 50  mg/ml | Mean | 0.346 | 0.356 | 0.343 | 0.376 | 0.351 | 0.384 | 0.358 | 0.392 | 0.371 | 0.403 | 0.377 | 0.424 |  |  |
|  | SE | 0.011 | 0.001 | 0.001 | 0.001 | 0.001 | 0.001 | 0.001 | 0.001 | 0.001 | 0.001 | 0.001 | 0.001 |  |  |
|  | SD | 0.039 | 0.001 | 0.002 | 0.002 | 0.002 | 0.001 | 0.001 | 0.001 | 0.002 | 0.002 | 0.002 | 0.002 |  |  |
| 75  mg/ml | Mean | 0.352 | 0.362 | 0.357 | 0.381 | 0.362 | 0.392 | 0.374 | 0.398 | 0.380 | 0.416 | 0.391 | 0.431 |  |  |
|  | SE | 0.001 | 0.001 | 0.001 | 0.001 | 0.001 | 0.001 | 0.001 | 0.001 | 0.001 | 0.001 | 0.001 | 0.001 |  |  |
|  | SD | 0.001 | 0.002 | 0.001 | 0.002 | 0.002 | 0.003 | 0.002 | 0.001 | 0.001 | 0.001 | 0.002 | 0.002 |  |  |
| 100mg/ml | Mean | 0.417 | 0.444 | 0.423 | 0.462 | 0.433 | 0.473 | 0.442 | 0.483 | 0.449 | 0.499 | 0.462 | 0.535 |  |  |
|  | SE | 0.001 | 0.001 | 0.001 | 0.001 | 0.001 | 0.001 | 0.001 | 0.001 | 0.001 | 0.001 | 0.001 | 0.001 |  |  |
|  | SD | 0.001 | 0.002 | 0.002 | 0.002 | 0.002 | 0.001 | 0.001 | 0.002 | 0.001 | 0.003 | 0.002 | 0.002 |  |  |

Appendix C

Mean, standard error and the standard deviation of *A. catechu* antioxidant activity

| Concentrations | | 5 minutes | | 10 minutes | | 15 minutes | | 20 minutes | | 25 minutes | | 30 minutes | | F  value | P value |
| --- | --- | --- | --- | --- | --- | --- | --- | --- | --- | --- | --- | --- | --- | --- | --- |
|  |  | Con | LoD | Con | LoD | Con | LoD | Con | LoD | Con | LoD | Con | LoD |  |  |
| 25  mg/ml | Mean | 0.487 | 0.515 | 0.516 | 0.523 | 0.526 | 0.539 | 0.533 | 0.573 | 0.547 | 0.588 | 0.549 | 0.599 | 45.528  E````````` | p≤0.05 |
|  | SE | 0.001 | 0.001 | 0.001 | 0.001 | 0.001 | 0.001 | 0.001 | 0.001 | 0.001 | 0.001 | 0.001 | 0.001 |  |  |
|  | SD | 0.001 | 0.001 | 0.002 | 0.002 | 0.001 | 0.001 | 0.001 | 0.002 | 0.001 | 0.002 | 0.002 | 0.002 |  |  |
| 50  mg/ml | Mean | 0.539 | 0.593 | 0.555 | 0.619 | 0.588 | 0.646 | 0.600 | 0.666 | 0.617 | 0.675 | 0.638 | 0.694 |  |  |
|  | SE | 0.001 | 0.001 | 0.001 | 0.001 | 0.001 | 0.001 | 0.001 | 0.001 | 0.001 | 0.001 | 0.001 | 0.001 |  |  |
|  | SD | 0.001 | 0.001 | 0.002 | 0.002 | 0.002 | 0.002 | 0.002 | 0.002 | 0.001 | 0.002 | 0.002 | 0.002 |  |  |
| 75  mg/ml | Mean | 0.634 | 0.687 | 0.673 | 0.730 | 0.689 | 0.743 | 0.700 | 0.756 | 0.716 | 0.772 | 0.722 | 0.783 |  |  |
|  | SE | 0.001 | 0.000 | 0.001 | 0.001 | 0.001 | 0.001 | 0.001 | 0.001 | 0.000 | 0.000 | 0.001 | 0.001 |  |  |
|  | SD | 0.002 | 0.002 | 0.002 | 0.002 | 0.002 | 0.002 | 0.002 | 0.002 | 0.001 | 0.001 | 0.002 | 0.004 |  |  |
| 100mg/ml | Mean | 0.684 | 0.722 | 0.693 | 0.752 | 0.713 | 0.763 | 0.723 | 0.772 | 0.735 | 0.788 | 0.744 | 0.807 |  |  |
|  | SE | 0.001 | 0.001 | 0.001 | 0.001 | 0.001 | 0.001 | 0.001 | 0.001 | 0.001 | 0.001 | 0.001 | 0.001 |  |  |
|  | SD | 0.002 | 0.002 | 0.002 | 0.002 | 0.002 | 0.002 | 0.001 | 0.002 | 0.002 | 0.001 | 0.002 | 0.002 |  |  |

Appendix D

Mean, standard error and the standard deviation of *P. minus* antioxidant activity

| Concentrations | | 5 minutes | | 10 minutes | | 15 minutes | | 20 minutes | | 25 minutes | | 30 minutes | | F  value | P value |
| --- | --- | --- | --- | --- | --- | --- | --- | --- | --- | --- | --- | --- | --- | --- | --- |
|  |  | Con | LoD | Con | LoD | Con | LoD | Con | LoD | Con | LoD | Con | LoD |  |  |
| 25  mg/ml | Mean | 0.355 | 0.443 | 0.365 | 0.456 | 0.385 | 0.466 | 0.396 | 0.486 | 0.415 | 0.497 | 0.424 | 0.507 | 24.299 | p≤0.05 |
|  | SE | 0.000 | 0.001 | 0.001 | 0.000 | 0.001 | 0.001 | 0.000 | 0.001 | 0.000 | 0.023 | 0.000 | 0.000 |  |  |
|  | SD | 0.001 | 0.002 | 0.002 | 0.001 | 0.004 | 0.002 | 0.001 | 0.002 | 0.001 | 0.079 | 0.001 | 0.002 |  |  |
| 50  mg/ml | Mean | 0.397 | 0.457 | 0.417 | 0.476 | 0.424 | 0.495 | 0.435 | 0.505 | 0.443 | 0.516 | 0.455 | 0.535 |  |  |
|  | SE | 0.000 | 0.000 | 0.000 | 0.000 | 0.001 | 0.000 | 0.000 | 0.000 | 0.001 | 0.001 | 0.001 | 0.001 |  |  |
|  | SD | 0.001 | 0.001 | 0.001 | 0.001 | 0.003 | 0.002 | 0.002 | 0.002 | 0.002 | 0.002 | 0.002 | 0.002 |  |  |
| 75  mg/ml | Mean | 0.506 | 0.585 | 0.517 | 0.594 | 0.534 | 0.603 | 0.546 | 0.618 | 0.555 | 0.634 | 0.566 | 0.644 |  |  |
|  | SE | 0.001 | 0.000 | 0.001 | 0.000 | 0.000 | 0.001 | 0.001 | 0.001 | 0.001 | 0.001 | 0.001 | 0.001 |  |  |
|  | SD | 0.002 | 0.002 | 0.002 | 0.002 | 0.002 | 0.002 | 0.003 | 0.003 | 0.002 | 0.002 | 0.002 | 0.002 |  |  |
| 100mg/ml | Mean | 0.663 | 0.727 | 0.675 | 0.735 | 0.687 | 0.754 | 0.704 | 0.764 | 0.715 | 0.776 | 0.726 | 0.795 |  |  |
|  | SE | 0.001 | 0.001 | 0.001 | 0.001 | 0.001 | 0.001 | 0.001 | 0.001 | 0.001 | 0.001 | 0.001 | 0.001 |  |  |
|  | SD | 0.002 | 0.004 | 0.003 | 0.004 | 0.002 | 0.002 | 0.002 | 0.002 | 0.002 | 0.002 | 0.002 | 0.003 |  |  |

Appendix E

Mean, standard error and the standard deviation of *S. polyanthum* antioxidant activity

| Concentrations | | 5 minutes | | 10 minutes | | 15 minutes | | 20 minutes | | 25 minutes | | 30 minutes | | F  value | P value |
| --- | --- | --- | --- | --- | --- | --- | --- | --- | --- | --- | --- | --- | --- | --- | --- |
|  |  | Con | LoD | Con | LoD | Con | LoD | Con | LoD | Con | LoD | Con | LoD |  |  |
| 25  mg/ml | Mean | 0.256 | 0.284 | 0.267 | 0.307 | 0.284 | 0.316 | 0.291 | 0.335 | 0.304 | 0.345 | 0.321 | 0.354 | 20.366 | p≤0.05 |
|  | SE | 0.001 | 0.001 | 0.001 | 0.001 | 0.000 | 0.001 | 0.001 | 0.001 | 0.001 | 0.001 | 0.001 | 0.001 |  |  |
|  | SD | 0.002 | 0.002 | 0.002 | 0.002 | 0.001 | 0.002 | 0.002 | 0.002 | 0.002 | 0.002 | 0.003 | 0.003 |  |  |
| 50  mg/ml | Mean | 0.315 | 0.395 | 0.335 | 0.405 | 0.354 | 0.422 | 0.363 | 0.434 | 0.374 | 0.444 | 0.394 | 0.455 |  |  |
|  | SE | 0.001 | 0.001 | 0.001 | 0.001 | 0.001 | 0.001 | 0.001 | 0.001 | 0.001 | 0.001 | 0.001 | 0.001 |  |  |
|  | SD | 0.002 | 0.002 | 0.002 | 0.003 | 0.002 | 0.003 | 0.003 | 0.002 | 0.002 | 0.002 | 0.003 | 0.002 |  |  |
| 75  mg/ml | Mean | 0.417 | 0.534 | 0.426 | 0.544 | 0.445 | 0.555 | 0.455 | 0.573 | 0.473 | 0.585 | 0.485 | 0.6 |  |  |
|  | SE | 0.001 | 0.001 | 0.001 | 0.001 | 0.001 | 0.001 | 0.001 | 0.001 | 0.000 | 0.001 | 0.001 | 0.001 |  |  |
|  | SD | 0.002 | 0.002 | 0.003 | 0.002 | 0.002 | 0.003 | 0.003 | 0.002 | 0.001 | 0.003 | 0.002 | 0.003 |  |  |
| 100mg/ml | Mean | 0.532 | 0.609 | 0.549 | 0.616 | 0.571 | 0.625 | 0.584 | 0.64 | 0.597 | 0.653 | 0.607 | 0.665 |  |  |
|  | SE | 0.001 | 0.001 | 0.001 | 0.001 | 0.001 | 0.001 | 0.001 | 0.001 | 0.001 | 0.001 | 0.000 | 0.001 |  |  |
|  | SD | 0.003 | 0.003 | 0.002 | 0.005 | 0.004 | 0.002 | 0.002 | 0.002 | 0.004 | 0.002 | 0.002 | 0.003 |  |  |
